# hYSK1 promotes cancer cell proliferation and migration through negative regulation of p16^INK4a^ under hypoxic conditions

**DOI:** 10.18632/oncotarget.21654

**Published:** 2017-10-06

**Authors:** Mee-Hyun Lee, Zigang Dong, Young-Joon Surh, Bu Young Choi

**Affiliations:** ^1^ China-US (Henan) Hormel Cancer Institute, Zhengzhou 450008, China; ^2^ Tumor Microenvironment Global Core Research Center, College of Pharmacy, Seoul National University, Seoul 08826, South Korea; ^3^ Research Institute of Pharmaceutical Sciences, College of Pharmacy, Seoul National University, Seoul 08826, South Korea; ^4^ Department of Molecular Medicine and Biopharmaceutical Sciences, Graduate School of Convergence Sciences and Technology, Seoul National University, Seoul 08826, South Korea; ^5^ Cancer Research Institute, Seoul National University, Seoul 110-744, South Korea; ^6^ The Hormel Institute, University of Minnesota, Austin, MN 55912, USA; ^7^ Department of Pharmaceutical Science & Engineering, Seowon University, Cheongju 28674, South Korea

**Keywords:** hYSK1, p16^INK4a^, MMP-2, tumor migration, hypoxia

## Abstract

The alteration of expression of p16^INK4a^, a well-known cyclin-dependent kinase inhibitor involved in cell cycle control, in tumors is unclear, especially under hypoxic conditions. To evaluate p16^INK4a^ regulation, we performed a protein microarray analysis. Among 1,800 proteins in the array, we identified hYSK1 as a novel protein that interacts with the tumor suppressor p16^INK4a^. hYSK1, a member of the Ste20 family of serine/threonine protein kinases, promotes cell migration and tumorigenesis and is activated by oxidative stress. However, the molecular mechanisms underlying the oncogenic potential of hYSK1 remain elusive. Here, we report that hYSK1 interacts with p16^INK4a^ under hypoxic conditions in tumors, where it negatively regulates p16^INK4a^, enhancing cancer cell migration. Hypoxic stimulation of hYSK1 reduces p16^INK4a^ accumulation through *p16* promoter regulation to interact with unphosporylated SP-1 and increases matrix metalloproteinase-2 (MMP-2) expression by activating the *MMP-2* promoter associated with cell migration and proliferation.Conversely, knocking down hYSK1 expression activated p16^INK4a^ expression and suppressed MMP-2 expression. Thus, hYSK1 is necessary as a trigger for inactivating p16^INK4a^ and activating *MMP-2* during tumor migration, suggesting that hYSK1 is a specific negative regulator of the tumor suppressor p16^INK4a^ and may represent a novel molecular target for reactivation of tumor suppressor genes in humans.

## INTRODUCTION

The cyclin/cyclin-dependent kinase (CDK) complexes play an important role in regulating cell cycle progression by phosphorylating specific proteins. Cyclin/CDK activities are modulated by both positive and negative regulators [1, 2]. Cyclin/CDK complexes control phosphorylation of the retinoblastoma protein (Rb) [3]. However, the changes in cyclins and CDKs under the hypoxic tumor microenvironment have not been clearly elucidated. CDK inhibitors (CDKIs) are negative regulators of cyclin/CDK complexes and comprise seven members, including the INK4 and Cip/Kip families. Interestingly, p16^INK4a^, a cell cycle inhibitor, is involved directly or indirectly in the inhibition of tumor formation. The p16^INK4a^ protein controls apoptosis [4, 5], cellular senescence [6, 7], and invasion through suppressing MMP-2 activity [8]. Additionally, overexpression of p16^INK4a^ decreases expression of the angiogenic factor, vascular endothelial growth factor (VEGF), in glioma cells [9]. However, the mRNA level and protein expression of p16^INK4a^ are reduced in tumors under hypoxic conditions, leading to a normal G1/S arrest [10]. However, the reason for reduced p16^INK4a^ transcript and/or protein levels in tumor hypoxia is unclear.

We performed protein microarray analysis to identify p16^INK4a^-interacting proteins. Among the interacting proteins, the highest binding affinity was shown by hYSK1. The human analog of YSK1 (hYSK1) is an oxidative stress response kinase induced 3- to 7-fold in response to reactive oxygen intermediates, but is not affected by other environmental stresses, growth factors, or cytokines [11]. hYSK1 targets the Golgi apparatus through the Golgi matrix protein, GM130. Activated hYSK1 phosphorylates 14-3-3ζ, which mediates protein transport, cell adhesion, and polarity for cell migration [12]. A low oxygen (O_2_) level or hypoxia is a characteristic feature of solid tumors [13]. During the progression of solid tumors towards malignancy, rapidly proliferating cancer cells receive an insufficient supply of growth factors, nutrients, and oxygen from the existing vasculature [14]. This results in an intratumoral hypoxic environment, which induces cell cycle arrest in some cancer cells [10]. In this study, we investigated the role of hYSK1 in regulating p16^INK4a^ during cell cycle progression under hypoxia. The results of the present study revealed that hYSK1 negatively regulates p16^INK4a^ as a representative suppressor of cell cycle progression in both the cytosol and nucleus under tumor hypoxic conditions. Notably hYSK1 interacts with p16^INK4a^, thereby blocking the nuclear translocation of p16^INK4a^ and subsequently regulates p16^INK4a^ gene expression by activating the transcription factor specificity protein-1 (SP-1) in the nucleus during mild hypoxia. We also found that the hYSK1/CDKI complex increases the promoter activity of *matrix metalloproteinase-2 (MMP-2)*, thereby enhancing the invasiveness and metastatic potential of cancer cells under hypoxic conditions.

## RESULTS

### p16^INK4a^ interacts with hYSK1

**Figure 1:**
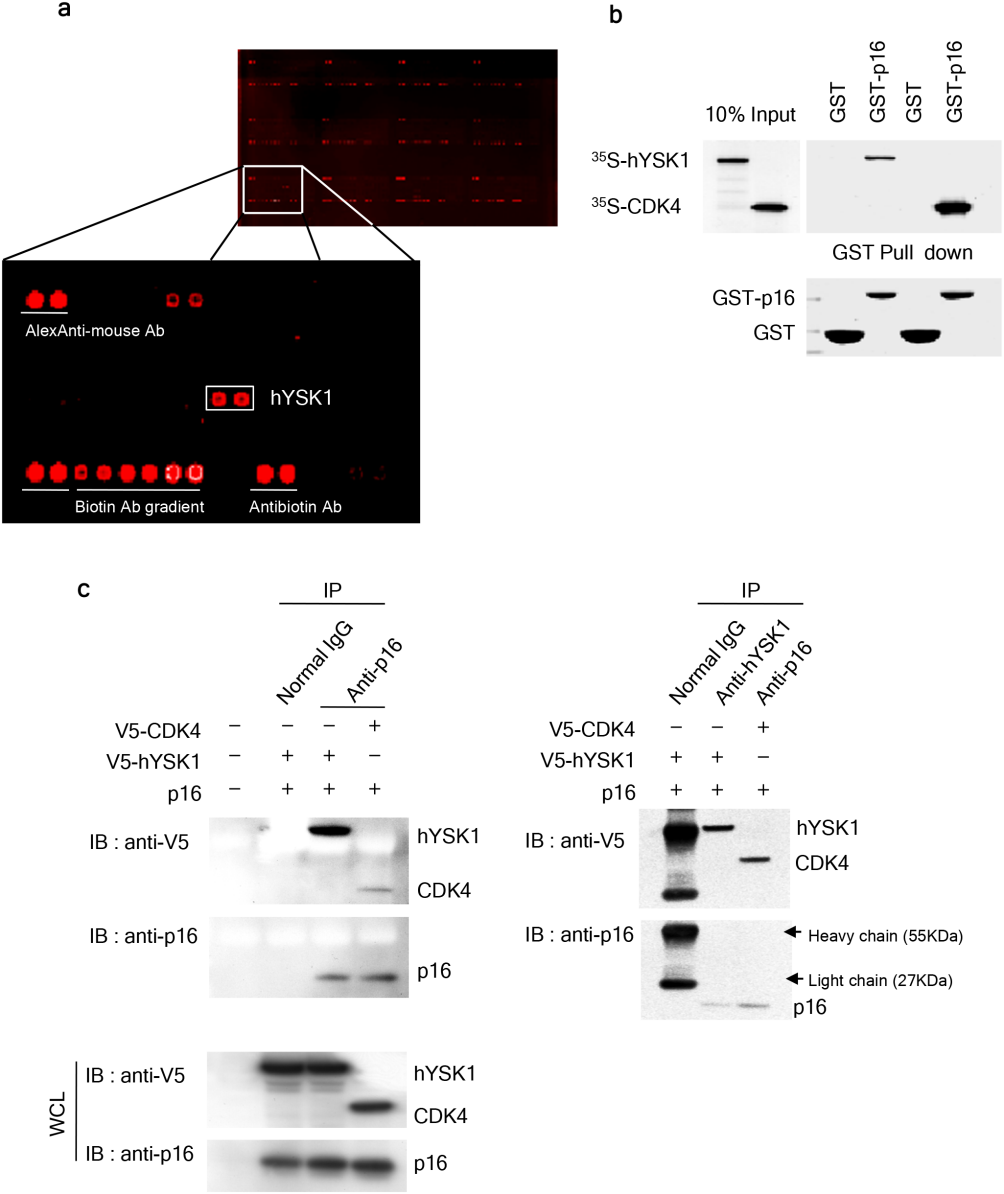
p16^INK4a^ interacts with hYSK1 **(a)** A human protein microarray was used to screen for novel binding partners of p16^INK4a^. **(b)** GST-p16^INK4a^ interacts with *in vitro* transcribed and translated ^35^S-met-hYSK1. ^35^S-met-CDK4, a well-known binding partner of p16^INK4a^, was used as a positive control. Western blots of *in vitro* translated and bound proteins are shown (upper panel). Coomassie blue-stained gels show the amount of GST–p16^INK4a^ and GST (as loading control) used in this assay (lower panel). **(c)** COS-1 cells were transfected with combinations of *p16*^*INK4a*^*, pcDNA3.1-V5-hYSK1*, and *pcDNA3.1-V5-CDK4.* Cell lysates were subjected to reciprocal immunoprecipitation and immunoblotting using anti-p16^INK4a^ (left panel) or anti-hYSK1 (right panel).

Reciprocal interaction studies form the basis of a powerful test to assess the ability of a protein to retain binding affinity while immobilized on a microarray surface [15]. We used protein arrays comprising 1,800 recombinant human proteins in order to identify the proteins that bind to the CDK inhibitor p16^INK4a^. We screened p16^INK4a^ interacting proteins using biotin-labeled p16^INK4a^. Laser scanning and digital image analysis demonstrated that p16^INK4a^ selectively and reproducibly interacted with hYSK1 (Figure 1a). To further confirm that hYSK1 directly interacts with p16^INK4a^, *in vitro* binding of hYSK1 and p16^INK4a^ was examined using a glutathione (GST) pull-down assay and co-immunoprecipitation. Full-length p16^INK4a^ proteins were produced in *Escherichia coli* BL21 as GST-fusion proteins and then purified with GST-Sepharose 4B beads. The ^35^S-labeled hYSK1 proteins were incubated with either GST or GST-p16^INK4a^ fusion proteins bound to Sepharose beads and precipitated in a pull-down assay. The results indicated that GST-p16^INK4a^ bound to hYSK1, whereas GST alone did not show any binding (Figure 1b). Proteins were extracted from COS-1 cells transiently transfected with *p16*^*INK4a*^*, pcDNA3.1-V5-hYSK1*, and *pcDNA3.1-V5-CDK4*, then immunoprecipitated with either p16^INK4a^ or hYSK1 antibody and immunoblotted with anti-V5 or anti-p16^INK4a^. The results indicated that hYSK1 was co-immunoprecipitated with p16^INK4a^ (Figure 1c, left panel) or reciprocally, p16^INK4a^ was co-immunoprecipitated with hYSK1 (Figure 1c, right panel). The interaction of p16^INK4a^ and CDK4 served as a positive control. These results suggest that p16^INK4a^ directly interacts with hYSK1.

### Identification of the hYSK1 and p16^INK4a^ binding site

**Figure 2:**
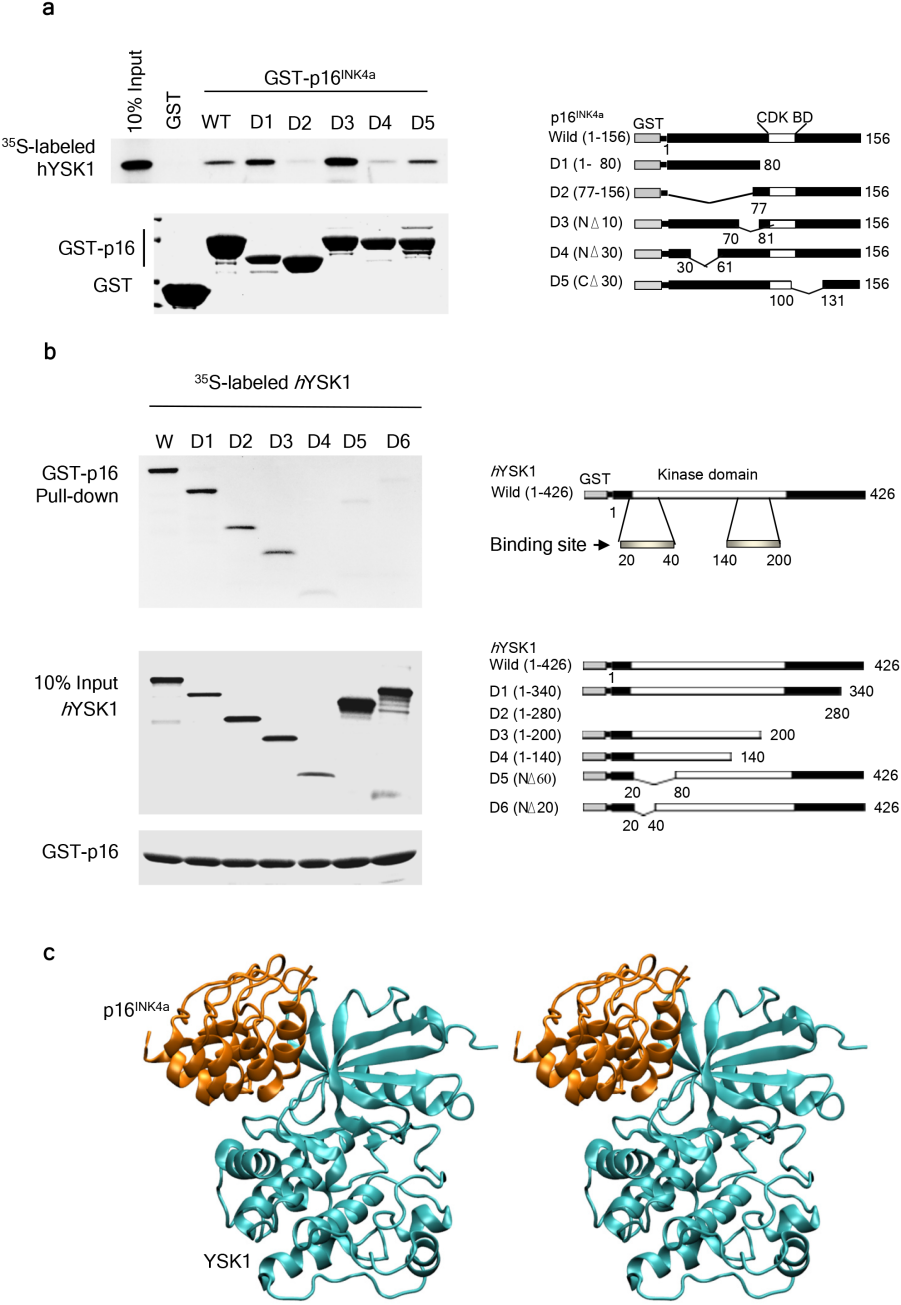
Identification of the binding domains of hYSK1 and p16^INK4a^ **(a)** Full length and deletion mutants of p16^INK4a^ were produced in *E. coli* BL21 as glutathione (GST)-fusion proteins and purified on GST-Sepharose 4B beads. ^35^S-labeled hYSK1 proteins were incubated with either GST or GST-p16^INK4a^ fusion proteins bound to Sepharose beads and were precipitated by GST pull-down. **(b)** Full-length and deletion mutants of hYSK1 were produced by *in vitro* transcription and translation. ^35^S-labeled hYSK1 proteins were incubated with GST-p16^INK4a^ fusion proteins bound to Sepharose beads and were precipitated by GST pull-down. Proteins bound to the beads were analyzed by autoradiography. Coomassie blue-stained gels were used in the assay as in Figure 1. **(c)** Predicted model of the p16^INK4a^ and hYSK1 complex generated by computer modeling.

To identify the specific binding regions of hYSK1 and p16^INK4a^, we generated deletion constructs of p16^INK4a^ and hYSK1 and then examined the binding of different domains of p16^INK4a^ with the full-length hYSK1 produced by *in vitro* transcription and translation*.* GST pull-down assay results showed that the D4 domain of p16^INK4a^, in which 30–60 amino acids were deleted, interacted with the full-length hYSK1 (Figure 2a). We also investigated binding of the full-length p16^INK4a^ to specific regions of hYSK1. Various deleted fragments of hYSK1 were expressed as *in vitro* transcribed and translated fusion proteins and their possible interaction with the full-length GST-p16^INK4a^ fusion protein was analyzed. GST pull-down assay revealed that the full-length GST-p16^INK4a^ interacted with the deleted fragment (residues 20-40 and 140-200) of hYSK1 (Figure 2b). These results indicated that the amino acids 30-60 of p16^INK4a^ and the amino acids 20-40 and 140-200 of hYSK1 are important binding sites involved in the interaction between hYSK1 and p16^INK4a^. We also performed a prediction analysis of the p16^INK4a^ and hYSK1 complex using the Fast Fourier transform-based docking algorithm ZDOCK (Figure 2c). The p16^INK4a^ and hYSK1 complex obtained from the protein-protein docking model suggested that p16^INK4a^ bound to the back of the hYSK1 kinase domain, which is located on the opposite side of the ATP binding pocket (Figure 2c). The hydrogen bond networks between the β-strands in the N-terminal domain of hYSK1 and the first, second, and third ankyrin repeats of p16^INK4a^ are expected to be crucial in the p16^INK4a^ and hYSK1 binding (Supplementary Table 1 and Figure 1). For example, the guanidinium side chain of Arg24 in p16^INK4a^ forms a pair of hydrogen bonds with the carboxylate oxygens of Asp24 in hYSK1. Moreover, Ala21, Tyr44, Arg46, Thr79, and Arg87 in p16^INK4a^ form hydrogen bonds with Lys36, Glu17, His41, Lys43, and Glu44 in hYSK1, respectively. In addition, the hydrophobic packing among the Ala21, Tyr44, Val51, and Met53 residues of p16^INK4a^ together with the Leu23, Ile38, Lys43, and Val45 residues of hYSK1 also contribute to the stability of the p16^INK4a^ and hYSK1 complex (Supplementary Figure 1). These hydrogen bonds and hydrophobic interactions might explain the *in vitro* and *in vivo* experimental results, which indicated that the segment between Ala20 and Ala60 of p16^INK4a^ and the segment between Phe20 and Ile50 of hYSK1 are the main binding segments of these proteins.

### hYSK1 inhibits the expression and translocation of p16^INK4a^ under hypoxic conditions

**Figure 3:**
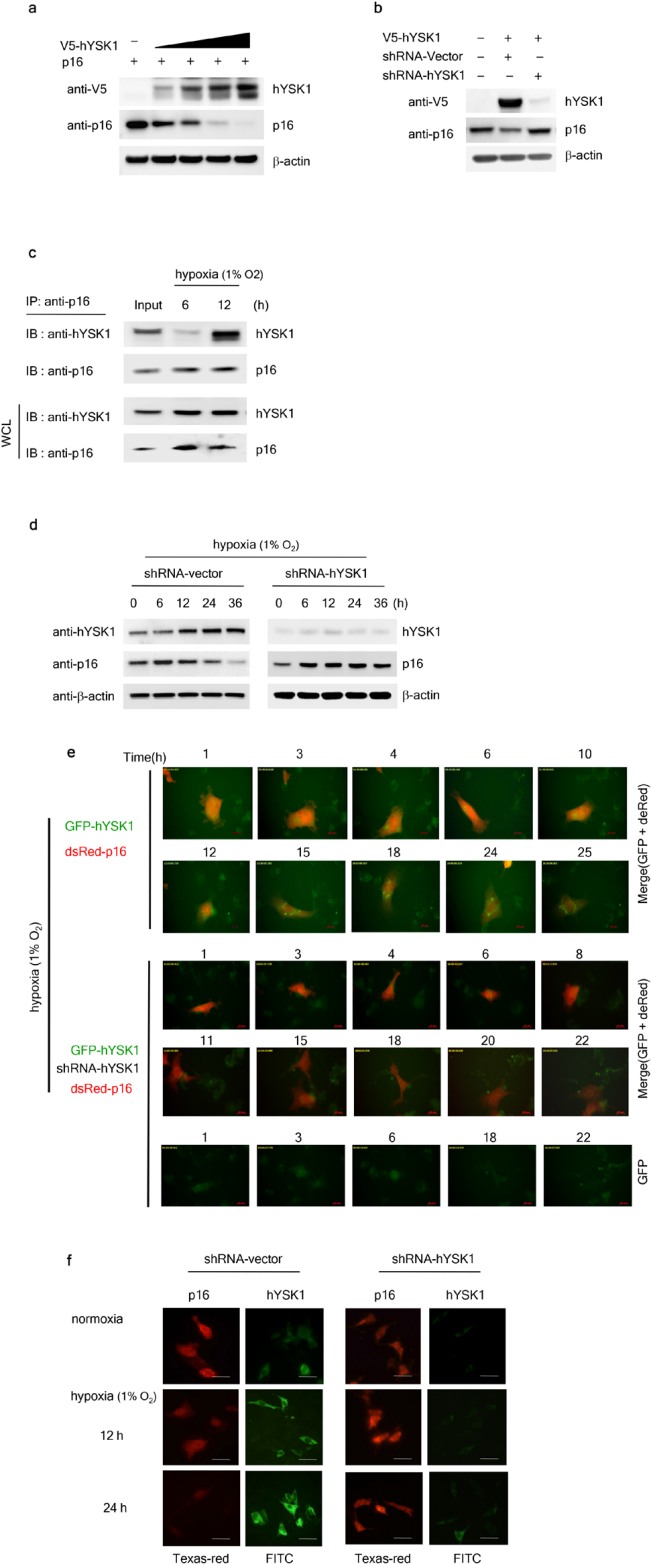
hYSK1 regulates expression and translocation of p16^INK4a^ **(a)** The effect of hYSK1 on p16^INK4a^ expression was determined by transfection of *pcDNA3.1-V5-hYSK1* in SK-MEL-28 cells. **(b)** Knocking down hYSK1 abundancerestored the expression of p16^INK4a^. **(c)** Immunoprecipitation was performed using anti-p16^INK4a^ in cells incubated under hypoxic (1% O_2_) conditions. p16^INK4a^ was immunoprecipitated followed by western blotting with anti-hYSK1 and anti-p16. **(d)** Relative expression of hYSK1 and p16^INK4a^ was assessed under hypoxic (1% O_2_) conditions by western blot analysis in SK-MEL-28 cells transfected with *shRNA-control* or *shRNA-hYSK1* plasmid. **(e)** The effect of hYSK1 on subcellular distribution of p16^INK4a^ in HT-1080 cells transfected with a *GFP-hYSK1/pDsRed-p16*^*INK4a*^ or *GFP-hYSK1/shRNA-hYSK1/pDsRed-p16*^*INK4a*^ plasmid was examined. Transfected cells were incubated under hypoxic (1% O_2_) conditions were examined by live cell imaging. **(f)** Time-dependent fluorescence localization of p16^INK4a^ (red) and hYSK1 (green) was visualized at 12 or 24 h after transfection of *shRNA-control* or *shRNA-hYSK1* in SK-MEL-28 cells both in normoxic and hypoxic (1% O_2_) conditions.

To examine the role of hYSK1 in the regulation of p16^INK4a^, we detected the effect of the ectopic expression or knockdown of hYSK1 on the expression level of p16^INK4a^. The amount-dependent over-expression of hYSK1 decreased p16^INK4a^ expression in SK-MEL-28 melanoma cells (Figure 3a). Transient transfection of SK-MEL-28 cells with short-hairpin RNA designed against hYSK1 (*shRNA-hYSK1*) completely suppressed hYSK1 expression and restored the expression of p16^INK4a^ (Figure 3b). These results suggest that hYSK1 expression might play an important role in the regulation of p16^INK4a^. In order to determine whether endogenous hYSK1 and p16^INK4a^ also interacted in cells, we performed immunoprecipitation using a p16^INK4a^ antibody followed by immunoblot analysis with a hYSK1 antibody as described in the Methods section. The p16^INK4a^ protein strongly interacted with hYSK1 at 12 h after exposure to hypoxia (1% O_2_; Figure 3c). SK-MEL-28 cells were transiently transfected with *shRNA-control* or *shRNA-hYSK1* and endogenous expression of hYSK1 under hypoxic (1% O_2_) conditions was then assessed after incubation for 36 h. Results showed that the *shRNA-hYSK1* completely inhibited hYSK1 expression (Figure 3d). Upon exposure to hypoxia (1% O_2_), p16^INK4a^ expression was increased in a time-dependent manner in *shRNA-hYSK1*-transfected SK-MEL-28 cells compared to cells transfected with the *shRNA-control* (Figure 3d). To determine whether nuclear translocation of the active p16^INK4a^ protein was affected by hYSK1 expression, the distribution of exogenous and endogenous p16^INK4a^ and hYSK1 upon exposure to hypoxia (1% O_2_) was investigated. The expression of hYSK1 was very low in the cytoplasm and nucleus of HT-1080 and SK-MEL-28 cells during normoxia, but its expression was increased upon exposure to hypoxic conditions (Figure 3e, 3f). Interestingly, the nuclear expression of p16^INK4a^ was decreased in a time-dependent manner, whereas its expression in the cytoplasm was retained under hypoxia following hYSK1 expression (Figure 3e, 3f). If hYSK1 is a negative regulator of p16^INK4a^ expression and translocation in hypoxic conditions, hYSK1 knockdown might be expected to increase the hypoxia-induced p16^INK4a^ protein expression and translocation. Thus, we knocked down hYSK1 expression in HT-1080 and SK-MEL-28 cells to assess whether the expression and nuclear translocation of the p16^INK4a^ protein are inhibited. The expression of p16^INK4a^ was substantially increased after exposure to hypoxia in *shRNA-hYSK1*-transfected cells compared to that in cells expressing *shRNA-control* (Figure 3e, 3f).

**Figure 4:**
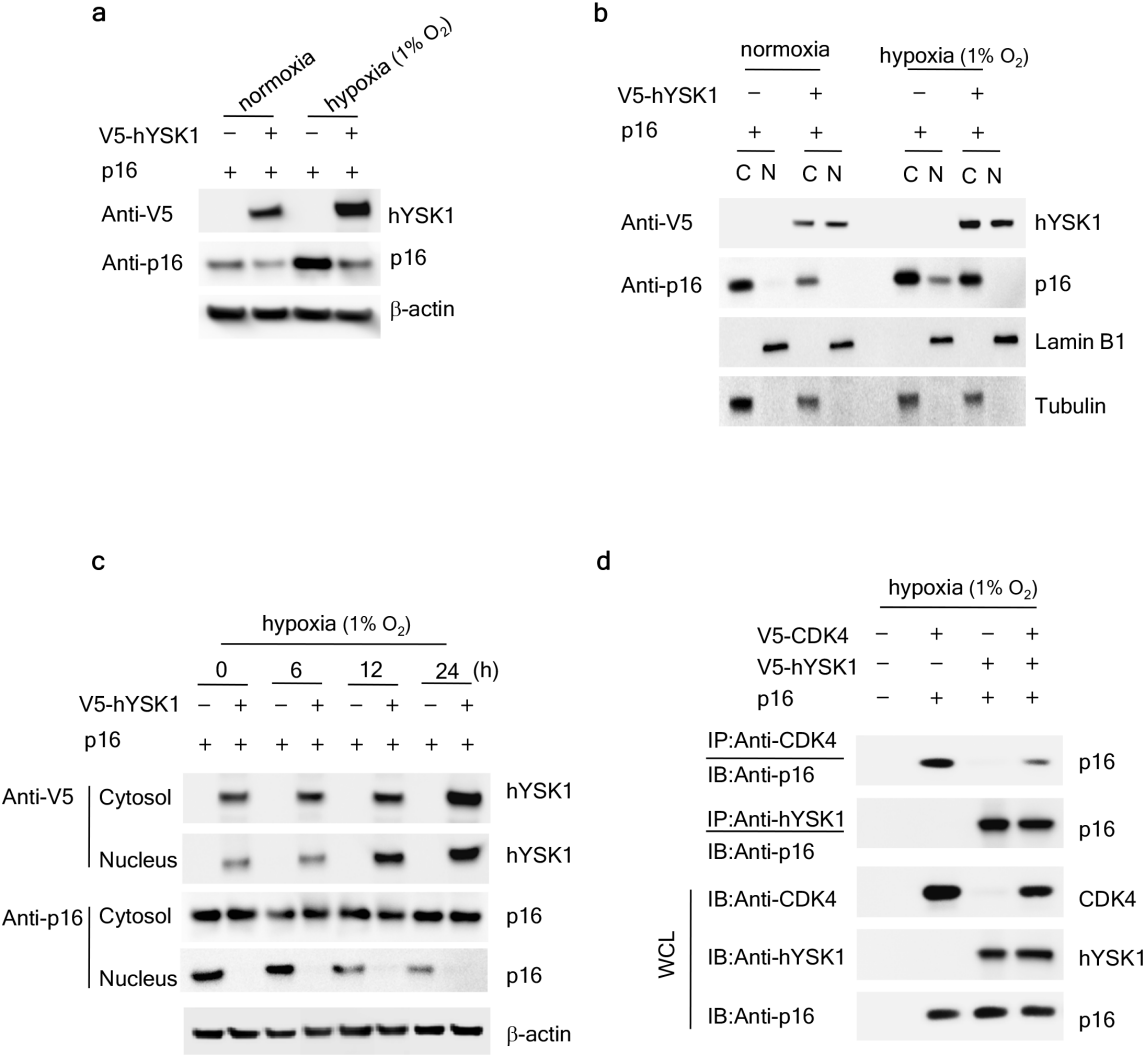
hYSK1 regulates the expression of p16^INK4a^ in the nucleus **(a)** The effect of hYSK1 on p16^INK4a^ expression was determined both in normoxic and hypoxic (1% O_2_) conditions after transfection of *pcDNA3.1-V5-hYSK1* and *pcDNA3.1-p16*^*INK4a*^ in SK-MEL-28 cells. **(b)** The cytosolic and nuclear expression of hYSK1 and p16^INK4a^ was examined after transfection of *pcDNA3.1-V5-hYSK1* and *pcDNA3.1-p16*^*INK4a*^ in SK-MEL-28 cells incubated under normoxic or hypoxic (1% O_2_) conditions. Lamin B1 and tubulin were used as loading controls for nuclear and cytosol fractions, respectively. **(c)** The expression of hYSK1 and p16^INK4a^ was examined both in the cytosol and nucleus at various time points under hypoxic conditions (1% O_2_). **(d)** Immunoprecipitation was performed using anti-CDK4 or anti-p16^INK4a^ in COS-7 cells exposed to hypoxic (1% O_2_) conditions. p16^INK4a^, hYSK1, and CDK4 in the lysates were co-precipitated and detected by western blotting.

To analyze the effect of hypoxia-induced hYSK1 activation on the expression of p16^INK4a^, we examined the effect of hYSK1 on p16^INK4a^ expression under normoxic or hypoxic (1% O_2_) conditions. The expression of p16^INK4a^ was downregulated with the ectopic expression of hYSK1 during both normoxia and hypoxia (Figure 4a). However, p16^INK4a^ expression was substantially elevated under hypoxic conditions in cells transfected with p16^INK4a^ alone and was downregulated in the presence of hYSK1. To investigate whether active hYSK1 can affect the nuclear localization of p16^INK4a^, the distribution of p16^INK4a^ and hYSK1 upon exposure to hypoxia was examined. Under normoxic conditions, hYSK1 was distributed both in the cytoplasm and nucleus in SK-MEL-28 cells, whereas p16^INK4a^ was localized in the cytoplasm (Figure 4b). Under hypoxic conditions, overexpressed p16^INK4a^ proteins were translocated from the cytoplasm to the nucleus. When hYSK1 and p16^INK4a^ were co-expressed in cells followed by exposure to hypoxia, hYSK1 attenuated the constitutive nuclear accumulation of p16^INK4a^ (Figure 4c). Hypoxia induced hYSK1 expression both in the cytoplasm and nucleus in a time-dependent manner and p16^INK4a^ was largely retained in the cytoplasm corresponding to a time-dependent decrease of its nuclear expression (Figure 4c). If hYSK1 functions as a critical regulator of the cell cycle and transactivation following p16^INK4a^ translocation during early hypoxia, hYSK1 might be expected to regulate the interaction of p16^INK4a^ with CDK4 for cell cycle arrest in hypoxia. Thus, to confirm the binding of p16^INK4a^ and CDK4, which might be influenced by hYSK1, COS-7 cells were transfected with p16^INK4a^ and CDK4 and/or hYSK1. Transfection was followed by co-immunoprecipitation with antibodies against CDK4, hYSK1, or p16^INK4a^ and subsequent immunoblot analysis (Figure 4d). Overexpression of p16^INK4a^ in the presence of either *V5-CDK4* or *V5-hYSK1* and subsequent immunoprecipitation with p16^INK4a^ revealed that p16^INK4a^ interacted with either CDK4 or hYSK1. However, in COS-7 cells transfected with p16^INK4a^ along with both *V5-CDK4* and *V5-hYSK1* and immunoprecipitated with anti-p16^INK4a^, p16^INK4a^ exhibited a stronger interaction with hYSK1 than with CDK4 (Figure 4d). These results indicate that hYSK1 negatively regulates the expression and nuclear localization of p16^INK4a^ by interfering with the interaction between p16^INK4a^ and CDK4. Therefore, we suggest that hYSK1 plays an important role as a p16^INK4a^ inhibitor *ex vivo.*

### hYSK1 inhibits p16^INK4a^ gene transcription and induces MMP-2 promoter activity; role of SP-1

**Figure 5:**
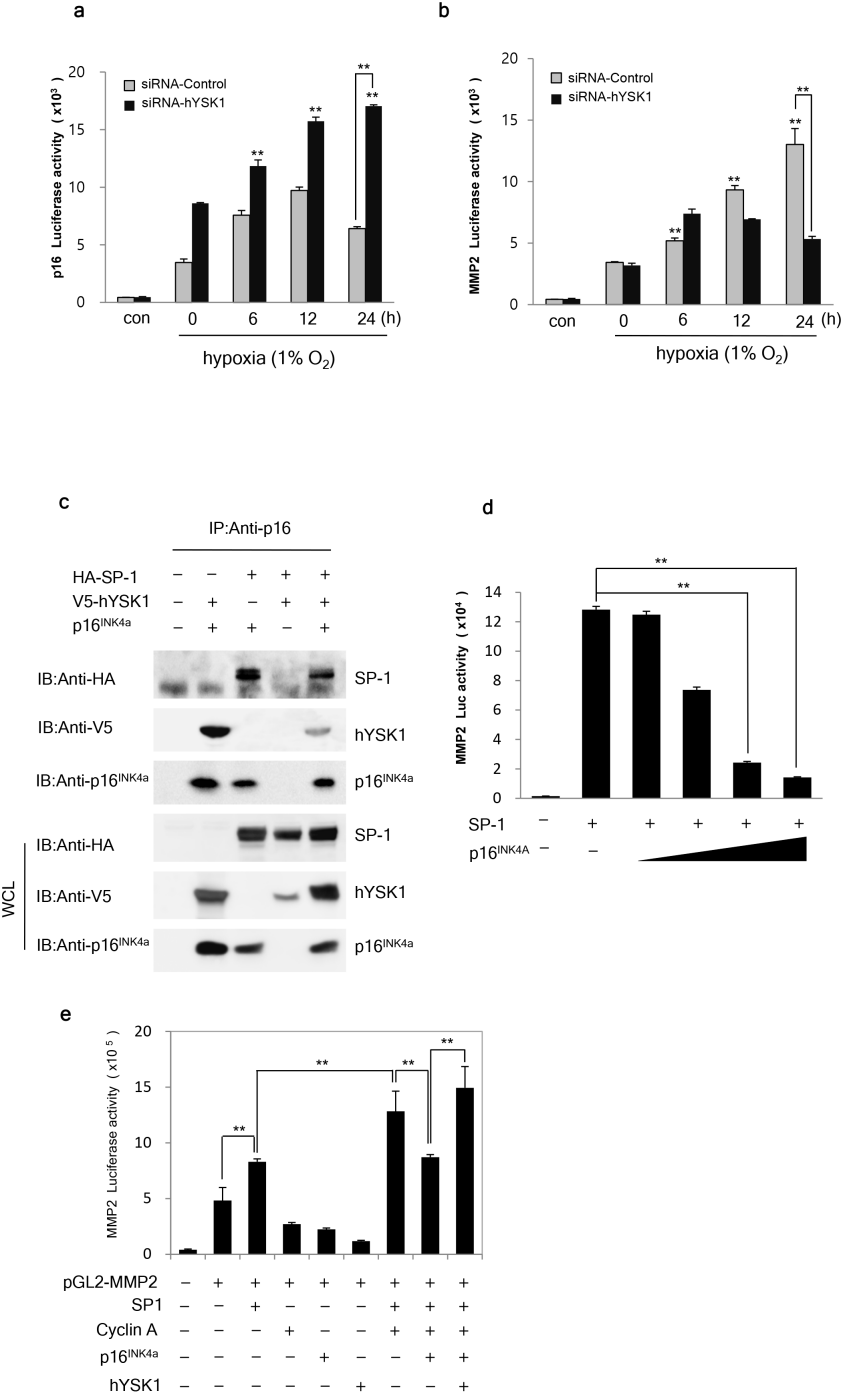
hYSK1 enhances the transcriptional activity of SP-1 transcription factor in the MMP-2 promoter through interfering p16^INK4a^ **(a)** A time course study of the silencing effect of hYSK1 on p16^INK4a^ luciferase activity in the SK-MEL-28 cell line under hypoxic conditions. **(b)** Time-dependent analysis of the silencing effect of hYSK1 on MMP-2 luciferase activity in the HT1080 cell line was examined under hypoxic conditions. **(c)** The interaction of hYSK1, SP-1 and p16^INK4a^ was detected by immunoprecipitation with a p16^INK4a^ antibody. **(d)** The effect of p16^INK4a^ on SP-1 activation of the transcription of MMP-2. **(e)** The SP-1 transcriptional activation of MMP-2 luciferase activity was assessed by various combinations of transfected SP-1, cyclin A, p16^INK4a^ or hYSK1. The expression of hYSK1, p16^INK4a^ and SP-1 were detected by western blot in SK-MEL-28 cells.

Under hypoxic conditions, gene transcription of p16^INK4a^ was increased until 12 h and then gradually declined until 24 h when cells were transfected with *shRNA-control*. However, in cells transfected with *shRNA-hYSK1*, the elevated gene transcription of p16^INK4a^ was sustained until 24 h (Figure 5a). These findings suggested that *shRNA-hYSK1*-transfected cells exhibited increased p16^INK4a^ gene transcription compared to *shRNA-control*-transfected cells at each time point.

Furthermore, unlike the MMP-2 activity under normoxic conditions, the MMP-2 gene transcription in *shRNA-hYSK1*-transfected cells was decreased from 12 h upon exposure to hypoxic conditions (Figure 5b). Moreover, COS-7 cells were transfected with *p16*^*INK4a*^*, V5-hYSK1*, or *HA-SP-1* and its cell lysates were immunoprecipitated with a p16^INK4a^ antibody. The results showed that p16^INK4a^ interacted with both hYSK1 and SP-1, forming a complex comprised of p16^INK4a^, hYSK1, and SP-1 (Figure 5c). Ectopic expression of increasing amounts of *p16*^*INK4a*^ in HT-1080 cells, which have a homozygous deletion of p16^INK4a^, resulted in decreased SP-1-dependent *MMP-2* expression (Figure 5d). Combinatorial transfection of the *MMP-2 reporter, SP-1, p16*^*INK4a*^*, cyclin A*, or *hYSK1* gene constructs resulted in increased *MMP-2* transcription in the presence of SP-1 and cyclin A. Its effect was significantly abolished by transfection with *p16*^*INK4a*^ and was restored in the presence of hYSK1 (Figure 5e). These results indicate that hYSK1 controls the expression of p16^INK4a^ and MMP-2 by regulation of SP-1 activity through masking p16^INK4a^ under hypoxic conditions.

### hYSK1 enhances proliferation and migration of cancer cells by downregulating p16^INK4a^

**Figure 6:**
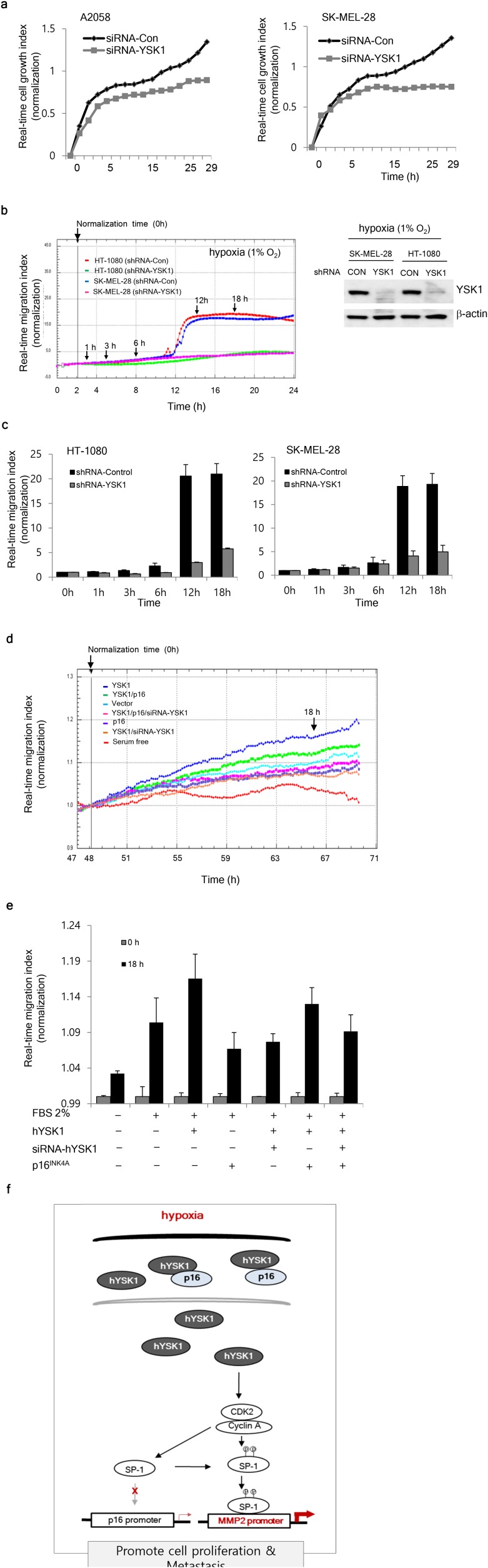
Physiologic effect of the interaction of hYSK1 and p16^INK4a^ in cancer cells **(a)** Growth of A5028 and SK-MEL-28 cells transfected with *si-hYSK1* was retarded compared to cells transfected with the *si-control* plasmid. **(b, c)** Under hypoxic conditions, the migration of HT-1080 and SK-MEL-28 cells was decreased in cells transfected with *shRNA-hYSK1* compared with cells transfected with *si-control*. **(d, e)** The effect of various transfection combinations on HT-1080 cancer cell migration. **(f)** A representative scheme of the regulation of p16^INK4a^ by hYSK1 in a hypoxic tumor. The increased expression of hYSK1 in hypoxic conditions retains cytosolic p16^INK4a^ and activates SP-1 transcriptional activity of the MMP-2 promoter. Thereby, cell proliferation and migration is promoted.

Next, we examined the physiological consequences of p16^INK4a^ regulation by hYSK1 in cancer cells. A2058 or SK-MEL-28 invasive melanoma cells transiently transfected with either *siRNA-control* or *siRNA-hYSK1* revealed that hYSK1 silencing reduced cell proliferation (Figure 6a). Moreover, migration of HT-1080 and SK-MEL-28 cells stably transfected with *shRNA-hYSK1* was dramatically reduced under hypoxic conditions compared to cells transfected with the *shRNA-control* (Figure 6b, 6c). To determine whether hYSK1 could activate cell migration through downregulation of *p16*^*INK4a*^, we performed co-transfection with various combinations of *hYSK1, p16*^*INK4a*^, or *siRNA-hYSK1* in HT-1080 cells. The results indicated that the migration index was increased by the addition of 2% serum under hypoxic (1% O_2_) conditions and that cell migration was further enhanced in *hYSK1*-transfected cells but was decreased in *p16*^*INK4a*^- and *hYSK1/siRNA-hYSK1*-transfected HT-1080 cells (Figure 6d, 6e). The decrease in migration index mediated by p16^INK4a^ was recovered by transfection with *hYSK1*. The hYSK1-induced recovery of p16^INK4a^-repressed cell migration was abrogated by transfection with *siRNA-hYSK1*. The cell migration enhanced by hYSK1 and its modulation by p16^INK4a^ was also examined using a wound healing assay with cells transfected using different combinations of *hYSK1, p16*^*INK4a*^, and *siRNA-hYSK1* (Supplementary Figure 2). We present a model (Figure 6d) depicting the interaction between hYSK1 and p16^INK4a^ and its consequences on cell proliferation and migration.

## DISCUSSION

*p16*^*INK4a*^, a tumor suppressor, is silenced by methylation in various cancers. Mutational inactivation of p16^INK4a^ has been shown to promote tumorigenesis [16]. The tumor suppressor function of p16^INK4a^ has been linked to its inhibition of CDK that leads to disassembly of the cyclin D1/CDK complex, thereby resulting in reduced cell proliferation [17, 18]. The growing tumor maintains an intratumoral hypoxic environment that stimulates the malignant progression of preneoplastic cells [19]. Expression of p16^INK4a^ is decreased in cancer cells under severe hypoxia [12]. Moreover, ectopic expression of p16^INK4a^ was reported to inhibit hypoxia-induced migration of the human breast cancer cells, MDA-MB231 [20]. However, the mechanisms underlying the loss of p16^INK4a^ function under hypoxic conditions and its role in the resultant increased proliferation and migration of cancer cells, remain elusive. In the present study, we sought to elucidate the inactivation mechanisms of p16^INK4a^ under tumor hypoxia and examine the effects of loss of p16^INK4a^ function on tumor progression.

Using protein microarray analysis, we identified hYSK1, a protein that facilitates cell migration [21], as an interacting partner of p16^INK4a^. Data from GST pull-down and immunoprecipitation assays further confirmed the interaction between hYSK1 and p16^INK4a^. Our study revealed that the D4 (30–60 amino acid) domain of p16^INK4a^ interacted with the 20–40 amino acid and 140–200 amino acid segments of hYSK1. Computational docking analysis indicated that the possible binding site of p16^INK4a^ would be located at the other side of the ATP binding pocket of hYSK1. We found that ectopic hYSK1 expression dose-dependently diminished the expression of p16^INK4a^, which was restored by silencing hYSK1. Moreover, the elevated hYSK1 expression in cells exposed to hypoxia showed a concomitant decrease in p16^INK4a^, which was further restored with hYSK1 knockdown. With hypoxic stimulation, hYSK1 interacted with p16^INK4a^ and retained p16^INK4a^ in the cytoplasm, thereby blocking its nuclear localization. These findings raised the possibility that the binding between hYSK1 and p16^INK4a^, and the subsequent inhibition of p16^INK4a^ expression might contribute to tumor progression. One of the mechanisms by which p16^INK4a^ inhibits tumor growth under hypoxic conditions is mediated through the interaction of p16^INK4a^ with CDK4 [8] and a subsequent decrease in CDK4 catalytic activity [18]. The finding that the interaction between p16^INK4a^ and CDK4 under hypoxic conditions is reduced in the presence of hYSK1 suggests that hYSK1 impairs the p16^INK4a^-mediated decrease in CDK4 activity and hence, promotes the growth of hypoxic tumor cells by sequestering p16^INK4a^ in the cytoplasm through protein-protein interaction.

To further elucidate the mechanisms involved in hYSK1 regulation of p16^INK4a^ and its consequences on cell migration, we first found that under hypoxic conditions, hYSK1 silencing increased the promoter activity of *p16*^*INK4a*^, but decreased the promoter activity of *MMP-2*. The p16^INK4a^ protein was reported to inhibit MMP-2 expression by inactivating SP-1 [22], which is a strong activator of p16^INK4a^ and p21^WAF1^ [23]. Because transactivation of both *p16*^*INK4a*^ and *MMP-2* promoters is regulated by SP-1, we focused on the impact of hYSK1 on the regulation of SP-1. However, immunoprecipitation of cells transfected with either *V5-hYSK1* or *HA-SP-1* with anti-hYSK1 or anti-SP-1 failed to detect either SP-1 or hYSK1, respectively. These findings suggest that hYSK1 does not interact with SP-1 directly. We therefore sought to examine the mechanisms underlying the negative and positive regulation of *p16*^*INK4a*^ and *MMP-2* promoter activity by hYSK1, respectively. SP-1 plays a key role in the transactivation of MMP-2 [22]. Haidweger et al. reported that SP-1 is phosphorylated by CDK2-cyclin A [5]. p16^INK4a^ negatively regulates MMP-2 expression by downregulating SP-1 function [22]. Thus, our finding that p16^INK4a^ diminished the SP-1 plus cyclin-A-dependent increase in *MMP-2* promoter activity, which was restored in the presence of hYSK1, suggests that the hYSK1 negation of p16^INK4a^ enhanced MMP-2 promoter activity. The increased *p16*^*INK4a*^ promoter activity and decreased *MMP-2* promoter activity in cells transfected with *siRNA-hYSK1* under hypoxic conditions confirmed the negative and positive regulation of p16^INK4a^ and *MMP-2* promoter activity by hYSK1. These findings were associated with decreased migration of different cancer cells transfected with *siRNA-hYSK1*.

Thus, our study reports the novel finding that inactivation of p16^INK4a^ is partly mediated through its binding with hYSK1, which sequesters p16^INK4a^ in the cytoplasm. The hYSK1-mediated inactivation of p16^INK4a^ results in elevated *MMP-2* promoter activity, which contributes to increased migration of cancer cells under hypoxia. Furthermore, a hYSK1 inhibitor might provide a new therapeutic window for the development of novel anticancer agents for activation of the tumor suppressor, p16^INK4a^.

## MATERIALS AND METHODS

### Plasmid construction

*pcDNA3.1-v5-hYSK1* and deletion fragments of hYSK1 were generated by PCR using the human cDNA clone-*hYSK1* (Origene Technologies, MD, USA) as a template. The PCR product was purified, digested with *EcoRI/XhoI*, and cloned into the *EcoRI/XhoI* sites of *pcDNA3.1-v5-HisA* (Invitrogen, MA, USA). For *TOPO-GFP-hYSK1*, the PCR product was inserted into the *pcDNA3.1-NT-GFP-TOPO* vector (Invitrogen, MA, USA). The GST-p16^INK4a^ and GST-deletion fragments of p16^INK4a^ were inserted in-frame into the *BamHI/XhoI* site of the *pGEX-5X-1* vector (Amersham Biosciences, PA, USA). For *pDsRed-p16*^*INK4a*^, the PCR product was cloned into the *ApeI* site of the *pDsRed* vector (Clontech Laboratories, CA, USA). *HA-SP-1* was cloned into the *EcoRI/XhoI* site of the *pCMV-HA* and *pCMV-Myc* vectors (Clontech Laboratories, CA, USA). The *shRNA-hYSK1* plasmid was constructed into the *pSilencer 4.1-CMV-hyg* vector (Ambion, NY, USA). The *pGL2-p16-luc* vector was a gift from Dr. Gordon Peters (Cancer Research UK, London) and the *pGL2-MMP-2-luc* vector was a gift from Dr. Etty N. Benveniste (The University of Alabama at Birmingham, Birmingham, AL). Various expression vectors were amplified in *E. coli* XL1-blue or BL21 cells and plasmids were purified using a Qiagen midi kit (Qiagen, Hilden, Germany). The DNA sequences of all plasmids were confirmed by sequencing (Dye Terminator ABI Type Seq., Bionex, NJ, USA)

### Cell culture and transfection

Human melanoma (SK-MEL-28, A2058), human fibrosarcoma (HT-1080), and COS-7 cells (African green monkey kidney) were purchased from the American Type Culture Collection (ATCC, VA, USA). SK-MEL-28, A2058, and HT-1080 cells were cultured in MEM containing penicillin (100 units/mL), streptomycin (100 μg/mL), sodium pyruvate (1 mM), and 10% fetal bovine serum (FBS) (Invitrogen, Gibco). COS-1 and COS-7 cells were grown in DMEM containing penicillin (100 units/mL), streptomycin (100 μg/mL), and 10% FBS. Cells were maintained at 37°C in a humidified atmosphere of 95% air/5% CO_2_. The *pcDNA3.1-p16*^*INK4a*^ or *pcDNA3.1-v5-hisA-hYSK* plasmid was transfected using the jetPEI poly transfection reagent (Polyplus) into HT-1080, COS-1, or COS-7 cells to generate p16^INK4a^- or p16^INK4a^-hYSK1-expressing cells. For transient gene silencing, *si-control* and *si-hYSK1* (Dharmacon, CO, USA) were transfected into HT-1080, SK-MEL-28, and A2058 cells. *shRNA-control* and *shRNA-hYSK1* were transfected into HT-1080 and SK-MEL-28 cells. For the reporter gene assay, COS-7 cells were seeded in 24-well plates and incubated for 24 h followed by transfection with *pcDNA3-p16*^*INK4a*^*, HA-SP-1, cyclin A, pGL2-p16-luc*, or *pGL2-MMP-2-luc* reporter plasmids using the jetPEI poly transfection reagent based on the manufacturer’s instructions. The *pRL-TK* reporter plasmid was used as an internal control. Cells were harvested after 24 h and disrupted with 5X lysis buffer. Luciferase activity was determined after normalization to *pRL-TK* activity (Promega, WI, USA). For immunofluorescence microscopy, SK-MEL-28 cells were transfected with *shRNA-control* or *shRNA-hYSK1* and incubated for 24 h. The cells were transferred to a hypoxic chamber and incubated for an additional 12 or 24 h. The cells were then washed with PBS and fixed with methanol for 5 min at room temperature. After blocking with 2% BSA in PBST for 10 min, cells were sequentially incubated with p16^INK4a^ and hYSK1 primary monocloncal antibodies followed by incubation with Texas-red and FITC-conjugated secondary antibodies.

### Protein microarray

To evaluate novel protein-protein interactions, a protein microarray was used as a powerful detection method [24]. The p16^INK4a^ protein was biotinylated and probed on a ProtoArray® Protein Microarray (v.1.0) printed on thin nitrocellulose slides (Promega, WI, USA). Briefly, the ProtoArray™ Human Protein Microarray slide was blocked and probed with biotin labeled p16^INK4a^. Proteins on the array were detected using streptavidin conjugated Alexa Fluor 647 and scanned for analysis (Invitrogen, MA, USA) after drying.

### Immunoprecipitation

Transfected COS-7 cells or SK-MEL-28 cells were harvested in NET-NL lysis buffer containing 50 mM Tris (pH 7.5), 5 mM EDTA, 150 mM NaCl, 1 mM DTT, 0.01% NP-40, 0.2 mM PMSF, and a mixture of protease inhibitors (Roche Diagnostics, Basel, Switzerland). Cell lysates (200 μg) were clarified by centrifugation before overnight incubation at 4°C with p16^INK4^ (Pharmingen, CA, USA), CDK4 (Cell Signaling, MA, USA), hYSK1, and SP-1 (Santa Cruz Biotechnology, TX, USA) antibodies in NET-NR buffer [50 mM Tris (pH 7.5), 5 mM EDTA, 150 mM NaCl, 1 mM DTT, 0.01% NP-40, 2 μg/mL BSA, 0.2 mM PMSF, and a mixture of protease inhibitors (Roche Diagnostics Basel, Switzerland)]. An aliquot of 50 μL pre-washed protein A/G-agarose beads (Roche Diagnostics; 50% slurry) was then added to the mixture and incubated for 2 h at 4°C. Immunoprecipitates were recovered by centrifugation, washed three times in NET-NW buffer [50 mM Tris (pH 7.5), 5 mM EDTA, 150 mM NaCl, 1 mM DTT, 0.01% NP-40, and 0.2 mM PMSF] and resolved by SDS-PAGE and western blotting.

### Western blot analysis

Cells were disrupted on ice for 30 min in cell lysis buffer [20 mM Tris (pH 7.5), 150 mM NaCl, 1 mM Na_2_EDTA, 1 mM EGTA, 1% Triton X-100, 2.5 mM sodium pyrophosphate, 1 mM β-glycerophosphate, 1 mM sodium vanadate, 1 μg/mL leupeptin, and 1 mM phenylmethylsulfonyl fluoride (PMSF)]. After centrifugation at 20,817 *g* for 15 min, the supernatant fraction was harvested as a total cellular protein extract. Protein concentration was then determined using a protein assay reagent (Bio-Rad labs, CA, USA). The total cellular protein extracts were separated by SDS-PAGE and transferred to polyvinylidene fluoride (PVDF) membranes in 20 mM Tris-HCl (pH 8.0), containing 150 mM glycine and 20% (v/v) methanol. Membranes were blocked with 5% nonfat dry milk in 1x TBS containing 0.05% Tween 20 (TBS-T) and incubated with antibodies against p16, YSK1, CDK4, SP-1, MMP-2, lamin B1, α-tubulin, and β-actin. Blots were washed thrice in 1 x TBS-T buffer followed by incubation with the appropriate HRP-linked IgG. The specific proteins in the blots were visualized using an enhanced chemiluminescence (ECL) detection kit (GE Healthcare Bioscience, PA, USA).

### GST pull-down assay

Full length and deletion mutants of hYSK1 or CDK4 were translated *in vitro* with L-[^35^S] methionine using the TNT Quick Coupled Transcription/Translation System (Promega, WI, USA). GST fusion proteins were collected on glutathione-Sepharose beads (GE Healthcare Bioscience, PA, USA) and incubated for 4 h at 4°C with ^35^S-Met-labeled hYSK1, its deletion mutants, or CDK4. The bound proteins were washed thrice and boiled with 2.5X sample buffer for 3 min, centrifuged, and the supernatant fraction was then examined by 15% SDS-PAGE analysis. Binding was detected by autoradiography.

### Protein-protein docking

Docking structures of p16^INK4a^ and YSK1 were generated using the rigid-body global search algorithm and further refinement by energy-minimization. Rigid-body docking was performed by the Fast Fourier Transform-Based Docking algorithm ZDOCK [25–27], where p16^INK4a^ and YSK1 were treated as solid objects. Subsequent energy-minimization and molecular dynamics simulations of the p16^INK4a^-YSK1 complex were performed using the Impact modules from the Schrödinger Software Suite [28, 29]. Details are provided in the Supplementary Materials.

### Fluorescence live cell imaging

To track individual hYSK1 and p16^INK4a^ expression, HT-1080 cells were transfected with *GFP-TOPO-hYSK1* and *pDsRed-p16*^*INK4a*^ or *shRNA-hYSK1*. The cells were incubated for 12 h in a 5% CO_2_ incubator and transferred to a hypoxic (1% CO_2_) chamber. The movement of cells was tracked every 8-10 min for 24 h at 16 fixed regions by an inverted microscope (BX50, Olympus, Tokyo, Japan). Cell movement was analyzed using AVI meta imaging software.

### Cell proliferation and migration assay

Proliferation of A2058 and SK-MEL-28 cells transfected with *si-control* or *si-hYSK1*, was assessed by determining the cell growth index using the xCELLigence RT-CES system and E plates (Roche Diagnostics Basel, Switzerland), which monitors the cellular events in real time by measuring electrical impedance across inter-digitized gold micro-electrodes integrated on the bottom of the culture plates. The impedance measurement indicates the quantitative value of cell number, viability, and morphology [30]. The cells were suspended in MEM containing 10% FBS and seeded in the E-plate 96 (2,000 cells/well/100 μl). The cells were monitored every 30 min for a period of 29 h. Wound migration of cells was measured using Culture-Inserts (Ibidi GmbH, Martinsried, Germany). The Culture-Inserts were placed in 6-well plates, and HT-1080 cells were seeded at a density of 5×10^4^ cells in each well with the Culture-Inserts. After 24 h of incubation, the Culture-Inserts were removed, and a cell-free gap of 500 μm was created. Phase contrast images of the closed gap were captured at 0 h (control) and 12 h of hypoxic incubation using an inverted microscope (magnification, 10X). For real-time cell migration, we used the xCELLigence RTCA DP system and fibronectin-coated CIM plates (Roche Diagnostics Basel, Switzerland) [31]. Cells were seeded at 10,000 cells per well and the migratory behavior of each cell line was monitored for 18 h. The assay was performed based on the manufacturer’s instructions (Roche Diagnostics Basel, Switzerland).

### Statistical analysis

Values were expressed as means ± S.E.M. from at least three independent experiments. Statistical significance was determined by Student’s *t*-test and a *p*-value less than 0.05 was considered statistically significant.

## SUPPLEMENTARY MATERIALS FIGURES AND TABLE


