# Supplementary material for: hYSK1 promotes cancer cell proliferation and migration through negative regulation of p16INK4a under hypoxic conditions
Source: Oncotarget. 2017 Oct 6;8(51):89072–85. doi: 10.18632/oncotarget.21654 (PMC5687670; doi:10.18632/oncotarget.21654)
Supplement: Supplementary file 1 [file oncotarget-08-89072-s001.pdf]

hYSK1 promotes cancer cell proliferation and migration through negative regulation of $p16^{INK4a}$ under hypoxic conditions

SUPPLEMENTARY MATERIALS

Protein-protein docking assay

We used 1.2Å as the grid spacing for translations and 6° as the angular step of rotational sampling of the smaller protein $p16^{INK4a}$ for the $p16^{INK4a}$-hYSK1 docking. Except for these parameters, all other default parameters were used. During the rigid-protein docking, pairwise shape complementarity, IFACE statistical potential and electrostatics were used to score protein-docking predictions [1–3]. ZDOCK program [1–3] outputted 2,000 sorted docked configuration possibilities and we selected the first 20 configurations with the lowest energies for further analysis. The selected binding mode ranked 2nd. To refine this crude structure of the $p16^{INK4a}$-hYSK1 complex using rigid-body docking, we first performed energy minimization with full flexibility of the complex using the Impref module from Maestro [4]. After this rigid protein-protein docking structure was minimized to remove the initial steric clashes, it was further refined by molecular dynamics (MD) simulations using the Impact module from the Schrödinger Software Suite [5]. During the molecular dynamics simulations, the Cα atoms of all residues from the $p16^{INK4a}$-hYSK1 complex were harmonic-constrained to prevent any large deviation of the protein backbone. Molecular dynamics simulations were performed with default options or modified settings. For the default options, constant temperature (NVT) simulations were run at room temperature with constant dielectric (dielectric constant 1.0), the OPLS_2005 force field, and the constraining force of 25 kcal/(mol Å) [2]. For the modified settings, we increased the number of MD steps from 100 to 10000 and a time step from 1 fs to 2 fs. As a result, a total of 20 ps simulation was performed. The final model of the $p16^{INK4a}$-hYSK1 complex is shown in Figure 1 and the refined protein-protein interactions were shown in Supplementary Figure 1 and Supplementary Table 1. The crystal structure of $p16^{INK4a}$ was obtained from the $p16^{INK4a}$-CDK6 complex solved at 3.4 Å resolution (PDB entry 1BI7 [6]) and that of Homo sapiens hYSK1 (residues 3-292; NCBI Accession No: D63780; Swiss-Prot Accession No: O00506) downloaded from the SWISS-MODEL Repository [7, 8]. This downloaded structure is a comparative homology model built on the basis of the crystal structure of yeast hYSK1 (PDB entry 2XIK, 1.97Å resolution).

REFERENCES

1. Mintseris J, Pierce B, Wiehe K, Anderson R, Chen R, Weng Z. Integrating statistical pair potentials into protein complex prediction. Proteins. 2007; 69:511-20.
2. Chen R, Weng Z. A novel shape complementarity scoring function for protein-protein docking. Proteins. 2003; 51:397-408.
3. Chen R, Li L, Weng Z. ZDOCK: an initial-stage protein-docking algorithm. Proteins. 2003; 52:80-7.
4. Schrödinger *Impact*. (L.L.C., New York, NY; 2010).
5. Schrödinger *Maestro v5.5*. (L.L.C., New York, NY; 2010).
6. Russo AA, Tong, L, Lee JO, Jeffrey PD, Pavletich NP. Structural basis for inhibition of the cyclin-dependent kinase Cdk6 by the tumour suppressor p16INK4a. Nature. 1998; 395:237-43.
7. Kiefer F, Arnold K, Kunzli M, Bordoli L, Schwede T. The SWISS-MODEL Repository and associated resources. Nucleic Acids Res. 2009; 37:D387-92.
8. Kopp J, Schwede T. The SWISS-MODEL repository of annotated three-dimensional protein structure homology models. Nucleic Acids Res. 2004; 32:D230-4.

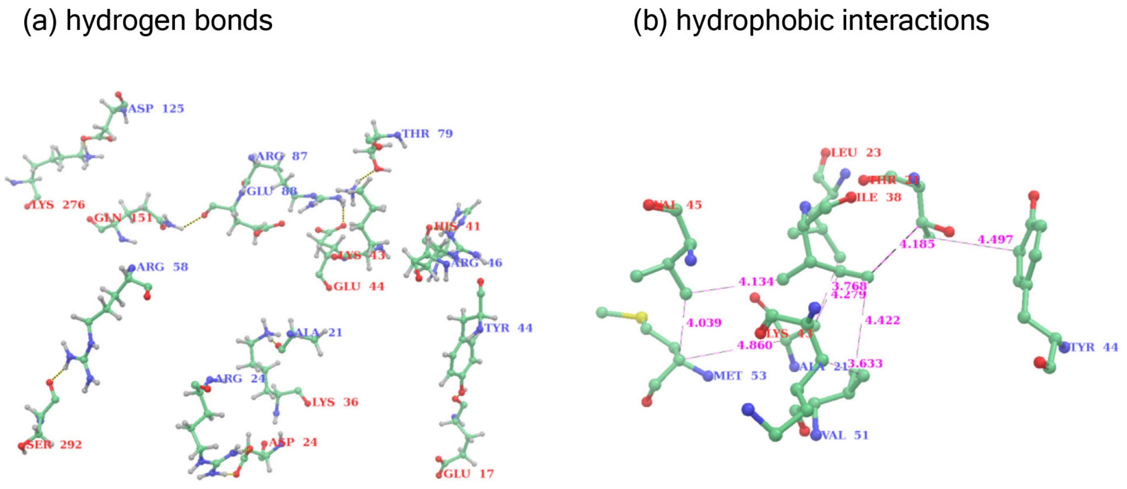

Supplementary Figure 1: Key interactions between interface residues in the computational model of the p16INK4a–hYSK1 complex. **(a)** 10 key hydrogen bonds. **(b)** hydrophobic interactions network. For a clear picture, only part of C-C interactions are listed here and all hydrogen atoms of hydrophobic packing residues are not shown**.** In both panels (a) and (b), the residues from the hYSK1 protein are labeled in red and those from $p16^{INK4a}$ in blue.

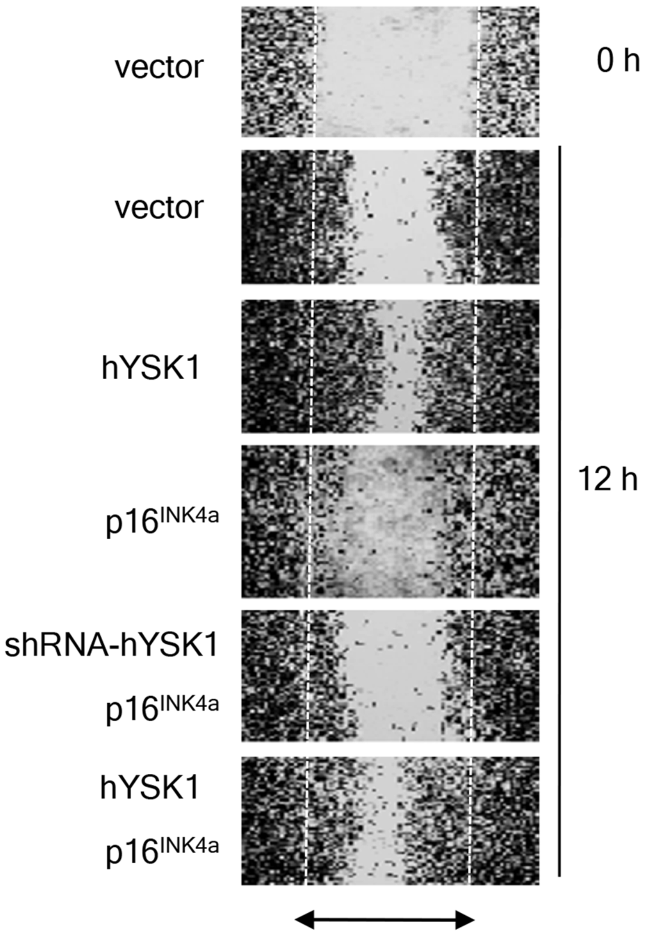

Supplementary Figure 2: The healing effect of hYSK1 in HT-1080 cells is increased by hindering p16INK4a. hYSK1 transfected cells promoted healing in wounded HT-1080 cells after 12 h and p16^{INK4a} or *shRNA-hYSK1/p16^{INK4a}* inhibited the process. Co-transfection of p16^{INK4a} and hYSK1 showed a healing effect similar to that of hYSK1-only transfected cells.

Supplementary Table 1: Hydrogen bonds in the computational model of the YSK1-p16INK4a complex

Element (Atom-H)	Element (Atom-A)	Distance (<2.5Å)	DHA angle (>120º)	HAB angle (>90º)
Ysk1: Lys36-HZ2	**P16(1st): Ala21-O**	1.514	170.1	167.9
**P16(1st): Arg24-HH12**	***Ysk1: Asp24-OD2***	1.739	170.6	122.8
**P16(1st): Arg24-HH22**	***Ysk1: Asp24-OD1***	1.669	154.0	109.0
**P16(2nd): Tyr44-HH**	Ysk1: Glu17-O	2.126	131.3	129.9
**P16(2nd): Arg46-HH12**	***Ysk1: His41-O***	2.398	123.3	117.7
**P16(2nd): Arg58-HH12**	Ysk1: Ser292-O	1.813	160.2	99.2
Ysk1: Lys43-HZ1	P16(3rd): Thr79-OG1	1.728	149.0	142.2
P16(3rd): Arg87-HH21	***Ysk1: Glu44-OE2***	1.808	144.1	115.5
Ysk1: Gln151-HE22	P16(3rd): Glu88-O	2.178	128.4	116.7
Ysk1:Lys276-HZ3	P16(4th): Asp125-OD2	1.707	145.9	102.2
